# Identification of *Fusarium solani* f. sp. *pisi* (*Fsp*) Responsive Genes in *Pisum sativum*

**DOI:** 10.3389/fgene.2020.00950

**Published:** 2020-08-18

**Authors:** Bruce A. Williamson-Benavides, Richard M. Sharpe, Grant Nelson, Eliane T. Bodah, Lyndon D. Porter, Amit Dhingra

**Affiliations:** ^1^Molecular Plant Sciences, Washington State University, Pullman, WA, United States; ^2^Department of Horticulture, Washington State University, Pullman, WA, United States; ^3^USDA-ARS, Grain Legume Genetics and Physiology Research Unit, Prosser, WA, United States

**Keywords:** pea, root rot, tolerance, susceptible, RNAseq, transcriptomics, gene expression, *Pisum sativum* L.

## Abstract

*Pisum sativum* (pea) is rapidly emerging as an inexpensive and significant contributor to the plant-derived protein market. Due to its nitrogen-fixation capability, short life cycle, and low water usage, pea is a useful cover-and-break crop that requires minimal external inputs. It is critical for sustainable agriculture and indispensable for future food security. Root rot in pea, caused by the fungal pathogen *Fusarium solani* f. sp. *pisi* (*Fsp*), can result in a 15–60% reduction in yield. It is urgent to understand the molecular basis of *Fsp* interaction in pea to develop root rot tolerant cultivars. A complementary genetics and gene expression approach was undertaken in this study to identify *Fsp*-responsive genes in four tolerant and four susceptible pea genotypes. Time course RNAseq was performed on both sets of genotypes after the *Fsp* challenge. Analysis of the transcriptome data resulted in the identification of 42,905 differentially expressed contigs (DECs). Interestingly, the vast majority of DECs were overexpressed in the susceptible genotypes at all sampling time points, rather than in the tolerant genotypes. Gene expression and GO enrichment analyses revealed genes coding for receptor-mediated endocytosis, sugar transporters, salicylic acid synthesis, and signaling, and cell death were overexpressed in the susceptible genotypes. In the tolerant genotypes, genes involved in exocytosis, and secretion by cell, the anthocyanin synthesis pathway, as well as the DRR230 gene, a pathogenesis-related (PR) gene, were overexpressed. The complementary genetic and RNAseq approach has yielded a set of potential genes that could be targeted for improved tolerance against root rot in *P. sativum*. *Fsp* challenge produced a futile transcriptomic response in the susceptible genotypes. This type of response is hypothesized to be related to the speed at which the pathogen infestation advances in the susceptible genotypes and the preexisting level of disease-preparedness in the tolerant genotypes.

## Introduction

The food industry contributes significantly to the world’s total greenhouse gas emissions (Poore and Nemecek, 2018). About 18% of the global greenhouse gas emissions are caused by livestock production, which supplies the majority of the dietary protein (Stehfest et al., 2009). Proposed mitigation efforts include a shift to plant-based protein as it is an environmentally sustainable option. The demand for plant-based protein is on the rise due to its health benefits (World Health Organization, 2015), as well as due to the ethical concerns related to exploiting animals as a source of protein (Johansson, 2019). The global plant-based protein market is expected to keep growing at a compound annual growth rate of 8.1% from 2019 to 2025 (Research and Markets, 2019). Popular plant-based meats from Impossible Foods and Beyond Meat have already reached some of the biggest food and retail brands in the United States.

Pea (*Pisum sativum* L.; Family Fabaceae) is a major contributor to this plant-derived protein market and has gained particular interest lately due to its high protein content (20–30%), especially lysine; overall high nutritional value; and relatively low cost (do Carmo et al., 2016; Peng et al., 2016; Xiong et al., 2018). The market for pea protein is expected to be $34.8 million in 2020 due to growing consumer interest in plant-based proteins as an alternative to animal-derived protein (Grand View Research, 2015; Pietrysiak et al., 2018). Pea also plays a critical role in sustainable agriculture due to its nitrogen-fixing capability, short life cycle, and low water usage; all of which make it a useful cover-and-break crop requiring minimal external inputs.

The United States is one of the world’s major pea producers. In the United States, harvested area of peas has increased by over 300% during the last 25 years; however, the yields have decreased an average of 7.5% throughout this timespan. This is likely due to the fact that the cultivars that were originally developed in the Pacific Northwest are being increasingly grown in less productive areas such as Montana and the Dakotas (Vandemark et al., 2014). Sustainable production of pea has been negatively affected by several diseases, predominantly root rots (Akhtar and Azam, 2014; Bodah et al., 2016). Root rots are the diseases of greatest impact to crop production worldwide (Kumari and Katoch, 2020). Frequently, root rot diseases involve more than one pathogen; therefore, the disease is known as root rot complex. Pathogens such as oomycetes and fungi are commonly involved in this root rot complex (Xu et al., 2012; Chittem et al., 2015; Gossen et al., 2016; Bodah, 2017).

One of the predominant causal agent of root rots in *P. sativum* is the soil fungus, *Fusarium solani* f. sp. *pisi* (*Fsp*). *F. solani* is a fungal soil-borne facultative parasite that is present worldwide (Zhang et al., 2006). The yields of *P. sativum* cultivars can be reduced by 15–60% by *Fsp* (Seaman, 1976; Grünwald et al., 2003; Porter et al., 2014). Over the years, hundreds of pea cultivars and germplasm core collections have been screened for *Fsp* resistance, and lines have been developed that demonstrate partial resistance to selected *Fsp* races (Coyne et al., 2008). An effort to identify tolerance to root rot in wild pea germplasm resulted in the identification of eight accessions with high levels of partial resistance (Porter, 2010). These accessions have been utilized for developing new cultivars. However, in tests replicated in the greenhouse and/or the field with derived selections, complete tolerance to *Fsp* has not been obtained (Grünwald et al., 2003; Porter et al., 2014; Bodah et al., 2016).

Understanding the genetic basis of tolerance to *Fsp* in a wide array of different pea breeding lines and cultivars has been pursued in several studies. The first QTL for *Fsp* tolerance was reported from a field study utilizing various parental lines that showed resistance to multiple root rots (Kraft, 1992; Feng et al., 2011). Recent studies conducted under controlled conditions have reported three QTLs; QTL *Fsp-Ps 2.1* explains 44.4–53.4% of the phenotypic variance within a 1.2 cM confidence interval. The other two QTLs, *Fsp-Ps 3.2*, and *Fsp-Ps 3.3* explain 3.6–4.6% of the phenotypic variance related to *Fsp* root rot tolerance (Coyne et al., 2015, 2019). While the genes underlying these QTLs have not yet been identified, there is a reason for optimism given the recent release of the pea reference genome (Kreplak et al., 2019). It is expected to facilitate the characterization of potential transcription factors, stress-associated phytohormone genes, Pathogenesis-related (PR) proteins, or pea phytoalexin Pisatin (Kendra and Hadwiger, 1984) in the interaction between pea and *Fsp*.

While genetic approaches for identifying disease tolerance or resistance genes are common, gene expression approaches to identify key genes in response to pathogen challenge remain scarce. A report of *Aphanomyces euteiches*-mediated root rot of pea was investigated using a gene expression approach, and novel genes responsive during the pathogenic interaction with *Medicago truncatula* were reported (Nyamsuren et al., 2003). Besides the expected induction of PR and defense genes, several novel genes were also reported to be overexpressed during the plant-pathogen interaction.

To the best of our knowledge, a gene expression approach to identify genes involved in *Fsp* tolerance in pea is yet to be reported. For gaining a comprehensive insight into the transcriptomic responses during the *Fsp* challenge, a comparative time-course RNAseq expression analysis was performed on four tolerant and four susceptible *P. sativum* genotypes that were selected from a preceding study (Bodah et al., 2016). Data analysis reaffirmed the role of Disease-Resistance Response 230 (DRR230) and sugar transporters, as well as expression patterns of genes associated with receptor-mediated endocytosis and exocytosis, cell death, and anthocyanin synthesis. Interestingly, several previously uncharacterized genes were also identified to be differentially expressed in both tolerant and susceptible genotypes, which may help illuminate the novel mechanism of pea-*Fsp* interaction.

## Materials and Methods

### Plant Material and *Fsp* Isolates

A total of eight, white-flowered pea genotypes were selected for pathogen challenge (Table 1). Four tolerant genotypes – 00-5001, 00-5003, 00-5004, and 00-5007 – were selected from the *Fsp* tolerant 5000 series (Porter et al., 2014). The 5000 series pea breeding lines were previously developed via single-seed descent from crosses initiated in a USDA–ARS greenhouse in Prosser, WA, in 1998. The parentage of 00-5001 is PH14-119/M7477// Coquette/3/86-2197/74-410-2 (Kraft, 1989; USDA–ARS NGRP, 2020). The parentage of 00-5003 is 69PH42-691004/Recette//Popet/3/PH14-119/DL-1/3/B563-429-2/PI257593//DSP TAC (USDA–ARS NGRP, 2020). The parentage of 00-5004 is 79-2022/ICI 1203-1//Menlo/3/PI189171/DL-2//75-786 (Kraft and Tuck, 1986; USDA–ARS NGRP, 2020). The parentage of 00-5007 is 00-5005/00-5006. 00-5005 parentage is B669-87-0/M7477//Blixt B5119/3/00-5001/74SN5/3/PH14-119/DL-1//74SN3/Recette/5/ FR-725 (Kraft and Giles, 1976; USDA–ARS NGRP, 2020). 00-5006 parentage is 00-5003/00-5004.

**TABLE 1:** Selected white-flowered pea genotypes for time-course transcriptome analysis in response to *Fusarium solani* f. sp. *pisi* (*Fsp*) challenge.

Genotype	Source^a^	*Fsp* tolerance level^b^	Other disease resistance^c^	100 seed weight	Leaf type^d^	Market Class
00-5001	USDA-ARS VFCRU	*	Fop races 1, 2 and 5	22.7	af	Green fresh
00-5003	USDA-ARS VFCRU	*	Fop races 1, 2 and 5	15.9	af	Green fresh
00-5004	USDA-ARS VFCRU	*	Fop races 1, 2 and 5	20.8	af	Green fresh
00-5007	USDA-ARS VFCRU	*	Fop races 1, 2 and 5	22.2	P	Green fresh
Aragorn’	ProGene	***	Fop races 1, 2; PSBMV	19.5	af	Green dry
Banner’	ProGene	***	Fop race 2, PM	18.7	af	Green dry
Bolero’	AsGrow	****	Fop race 1, PM, Pythium, EMV	20.12	P	Green fresh
DSP’	Canner Seed	***	–	20.9	P	Green fresh

The table summarizes pea genotypes, source, *Fsp* tolerance level, other disease resistance, 100 seed weight, leaf type, and market class. ^*a*^AsGrow = AsGrow Seed Co., San Juan Bautista, CA, United States; Canner Seed = Canner Seed Co., Idaho Falls, ID, United States; ProGene = ProGene LLC Plant Research, Othello, WA, United States; USDA-ARS VFCRU = USDA-ARS, Vegetable and Forage Crops Research Unit, Prosser, WA, United States. ^*b*^*Fsp* tolerance = *Fsp* tolerance resulted from phenotyping, ranging from most tolerant (*) to most susceptible (****) (Bodah et al., 2016). ^*c*^Fop = *Fusarium oxysporum*; PSBMV = Pea Seed-Borne Mosaic Virus, PM = Powdery Mildew; EMV = Enation Mosaic Virus. ^*d*^af (Afila) = semi-leafless; P (Perfection) = normal leaf type. It is a gradient scale where * is tolerant, ** is less tolerant, *** is susceptible, and **** represents completely susceptible.

Four susceptible genotypes – “Aragorn,” “Banner,” “Bolero,” and “DSP” – were identified among frequently used commercial pea varieties. These eight genotypes were selected based on their contrasting root disease severity index reported in a preceding study (Bodah et al., 2016). The eight genotypes were classified as either tolerant or susceptible post-*Fsp* challenge by phenotyping of the root disease severity index, plant height, shoot dry weight, and root dry weight (Bodah et al., 2016). The 5000 series pea breeding lines were found to be the most tolerant lines among the white-flowered pea lines.

The *Fsp* isolates Fs 02, Fs 07, and Fs 09, were obtained from infected pea roots collected in the Palouse Region of Washington and Idaho, United States soils by Dr. Lyndon Porter, USDA-ARS Vegetable and Forage Crops Research Unit, Prosser, WA. The three isolates were single-spored, identified as *Fsp*, based on partial translation elongation factor 1-a sequences using Fusarium-ID (Geiser et al., 2004), and their pathogenicity to pea was confirmed (Bodah et al., 2016). The three isolates were grown on pentachloronitrobenzene (PCNB) selective media for 6 days (Nash and Snyder, 1962). Cultures were transferred to KERR’s media (Kerr, 1963), and incubated on a shaker at 120 rpm under continuous light for 6 days at 23–25°C. The spore concentration of each isolate was determined using a hemocytometer and diluted to 1 × 10^6^ spores/ml of water. A spore suspension inoculum containing equal parts by volume of each of the three isolates was created.

### *Fsp* Disease Challenge

*Fusarium solani* f. sp. *pisi* disease challenge was performed as reported previously (Bodah et al., 2016). Briefly, seeds of each pea genotype were sterilized in a 0.6% sodium hypochlorite solution and rinsed in sterile distilled H_2_O. Seeds were then soaked for 16 h in either the *Fsp* spore suspension (inoculated set) or in sterile H_2_O (control set). At the end of 16 h, seeds from the control and inoculated set were separated from the respective solutions and maintained on perlite under ambient light and controlled greenhouse conditions with temperature ranging between 21 and 25°C for either 6- or 12-h duration. The 0-h time point was represented by the endpoint of the 16-h soaking period. The embryo containing region of the seed was excised and harvested from 600 seeds per genotype per time point (0, 6, and 12 h), immediately frozen under liquid nitrogen and transferred to storage at −80°C for subsequent RNA extraction. The experiment was repeated three times in tandem under identical conditions.

### RNA Isolation, cDNA Library Construction and Sequencing

The frozen seed material from two out of the three pathogen challenge experiments was randomly selected and pulverized in a SPEX SamplePrep 6870 FreezerMill (SPEX SamplePrep, Metuchen, NJ, United States) for five cycles. Each cycle consisted of cooling for 2 min and grinding at 15 counts per second for 4 min. Total RNA was isolated from the pulverized tissue using the RNeasy Plant RNA Extraction Kit (Qiagen, Hilden, Germany). A Nanodrop ND-8000 Spectrophotometer (Thermo Fisher Scientific, Waltham, MA, United States) and a Qubit Fluorometer (Life Technologies, Camarillo, CA, United States) were used to quantify the extracted RNA. Contaminating DNA was removed using the TURBO DNA-free Kit (Life Technologies, Camarillo, CA, United States) using the manufacturer’s instructions. RNA quality was verified via electrophoresis on a 1% agarose gel.

Equimolar amounts of RNA samples from tolerant and susceptible genotypes were bulked for each time point before the construction of RNAseq libraries. RNAseq libraries were constructed using 1 μg of RNA, and the Illumina TruSeq kits (Illumina Inc., San Diego, CA, United States). RNA was purified with an Oligo(dT) cellulose affinity matrix and subsequently fragmented into short pieces of an average size of 450 base pairs with Ampure XP beads (Beckman Coulter, Brea, CA, United States). All libraries were quantified on a Qubit Fluorometer (Life Technologies, Camarillo, CA, United States) and analyzed on an Agilent BioAnalyzer (Agilent Technologies, Santa Clara, CA, United States) to determine the concentration, final size, and purity of the library. A total of 24 libraries were sequenced using the HiSeq2000 configuration 100 PE (Illumina Inc., San Diego, CA, United States) at the Michigan State University Genomics core laboratory.

### RNAseq Data Processing and Statistical Analysis

The generated fastq files were analyzed for quality with CLC Bio Genomics Workbench 6.0.1 (CLC Bio, Aarhus, Denmark) and trimmed with trimmomatic (Bolger et al., 2014). *De novo* RNAseq assembly was performed using data from all 24 samples to obtain a master assembly with the software Trinity v2.8.4 (Grabherr et al., 2011). Dependencies for Trinity, Bowtie2 v1.2.3 (Langmead and Salzberg, 2012), Salmon v0.12.0 (Patro et al., 2017), and JELLYFISH v2.2.3 (Marçais and Kingsford, 2011), were used during assembly. Bowtie2 and Salmon were used for abundance estimation, and JELLYFISH was used as a k-mer counting software.

The software Kalisto was used for transcript quantification (Bray et al., 2016). The reads were quantified for each of the two biological replicates of the tolerant or susceptible genotypes at three time points, 0, 6, 12 h after inoculation, and for each control or treatment. This analysis resulted in 24 separate quantification groups that were used for comparison. Differentially expressed contigs (DECs) with *p*-value of <0.001 and a greater than two-fold change in expression were identified using the Baggerley’s test for data from each of the two biological replicates generated for each genotype group, time point, and inoculation treatment. The RPKM (Reads Per Kilobase of transcript per Million mapped reads) expression values were also ascertained for each contig. Heat maps showing fold-change of RPKM values between control (C) and inoculated (I) sets and among genotypes were created in Microsoft Excel 365 ProPlus (Microsoft Corporation, Redmond, WA, United States).

### Functional Annotation, Statistical Gene Ontology (GO) Enrichment, and Pathway Analysis

Functional annotation of the master assembly and DECs was conducted via BLAST in BLAST2GO v. 3.3. (Conesa and Götz, 2008). Default parameters were used for the functional annotation, as well as for Gene ontology (GO) mapping, and InterProScan. The two-tailed Fisher’s exact test (FDR < 0.05) was used to ascertain over- and under-represented functions during the *Fsp* challenge. A heat map representing “biological process” GO terms over-represented in the *Fsp* inoculated treatment was created in Microsoft Excel 365 ProPlus (Microsoft Corporation, Redmond, WA, United States). The Kyoto Encyclopedia of Genes and Genomes (KEGG) pathway analysis was performed to identify pathways represented by the set of DECs for each time point and genotype.

### Real-Time Quantitative PCR

RNA was extracted utilizing the RNeasy Plant DNA Extraction Kit (Qiagen, Mainz, Germany) from the same sampled tissues utilized for the RNAseq analysis. After DNase treatment, equimolar amounts of RNA from the tolerant and susceptible genotypes were bulked for each time point. First-strand cDNA synthesis was performed using 1,500 ng of each bulked RNA sample with the SuperScript Vilo kit (Thermo Fisher Scientific, Waltham, MA, United States). Nine genes were randomly selected from the list of DECs for RT-qPCR analysis (Supplementary Table S1). Primers for RT-qPCR were designed with the Primer3 software (Rozen and Skaletsky, 2000) with the corresponding transcriptome contig as the query sequence for each primer set. The *P. sativum* root border cell-specific protein (GenBank accession AF1139187.1) was used as an internal reference control as it showed invariant expression across genotypes and treatments in the RNAseq data.

The QUBIT 3.0 fluorometer (Invitrogen, Carlsbad, CA, United States) was used to quantify cDNA library concentration. For each reaction, 16 ng of cDNA was used with the iTaq Universal SYBR Green Supermix (Bio-Rad, Berkeley, CA, United States). Each RT-qPCR reaction was performed in triplicate for each of the three biological replicates using the Stratagene M×3005P (Thermo Fisher Scientific, Waltham, MA, United States). The amplification profile consisted of an initial denaturation at 95°C for 150 s, 40 cycles of 20 s at 95°C for denaturation, 20 s at 60°C for annealing, and 20 s at 72°C for extension. A melting curve analysis was performed post amplification to ensure the presence of a unique amplicon and performed with an initial denaturation at 95°C for 1 min and a decrease of temperature to 50°C for annealing. The temperature was then increased in 0.5°C increments at 5 s/step from 50 to 95°C for fluorescence readings. Raw fluorescence data was used as input for crossover threshold (Ct) calculations and reaction efficiencies adjusted with LinRegPCR 2012.0 software (Ruijter et al., 2009). The ΔΔCt method offered by PE Applied Biosystems (Perkin Elmer, Forster City, CA, United States) was used to obtain relative differential expression values after reaction efficiencies were adjusted with the LinRegPCR 2012.0 software (Pfaffl, 2001).

### Functional Annotation of QTL Associated With *Fsp* Tolerance in Pea

*Fsp-Ps 2.1*, the major QTL found to be associated with *Fsp* tolerance in pea (Coyne et al., 2019), was annotated using the transcriptome data generated in this study to determine if there are any differentially expressed genes located in the selected genomic region. *Fsp-Ps 2.1* explains 44.4–53.4% of the phenotypic variance, and it is located on chromosome II within a 1.2 cM confidence interval of marker Ps900203 (Duarte et al., 2014; Coyne et al., 2019). The genomic sequence of the *Fsp-Ps2.1* ± 1.2 cM (±201,800 nt) was obtained from the pea genome (Kreplak et al., 2019). The length of the QTL sequence was calculated based on the distance between the Ps900203 and Ps000075 markers. Marker Ps000075 is 0.6 cM = 168,166 nt away from Ps900203. The transcriptome data from this study was aligned via BLAST against the *Fsp-Ps2.1* ± 1.2 cM sequence in CLC Bio Genomics Workbench 6.0.1 (CLC Bio, Aarhus, Denmark).

## Results

### Assembly of Transcriptome Data and Identification of Differentially Expressed Contigs (DECs)

A total of 850 million reads were generated after the sequencing of the 24 libraries (Supplementary Table S2). For these 24 libraries, the mean Q score ranged from 34.00 to 34.88; these Q scores validate the quality of the assay (Supplementary Table S2). After QC and trimming of low-quality reads, 69.49% of reads were used for assembly of the master transcriptome. The master transcriptome, generated in this study, was composed of 185,721 contigs (Supplementary Table S3), which had a mean contig length of 1503.15 nucleotides (nt) with a length range of 184–18,990 nt.

Mapping of reads to the master assembly showed a different number of contigs with zero mapped reads for each time point (0, 6, 12 h) and genotype (tolerant and susceptible). After expunging contigs with zero mapped reads, the total number of contigs ranged from 102,382 to 141,530, or 55.13 to 76.21% of the total 185,721 contigs, respectively (Supplementary Table S2). The total number of reads mapped to each contig for each time point and genotype and RPKM values are summarized in Supplementary Table S3.

**FIGURE 1:**
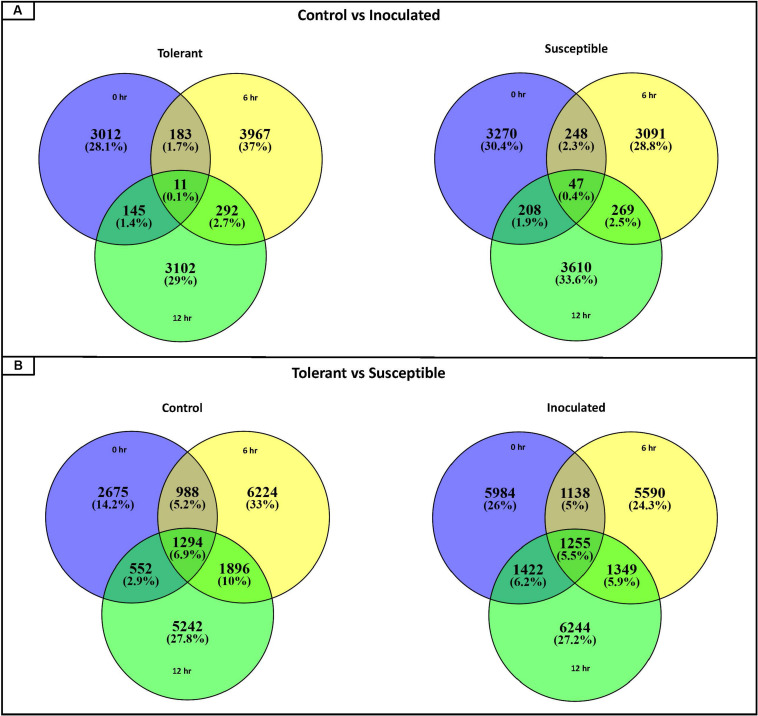
Venn diagrams representing the number of DECs (Differentially Expressed Contigs) for the 12 pairwise comparisons. **(A)** Number of DECs for pairwise comparisons between control and inoculated samples collected at 0, 6, and 12-h time points for the tolerant and the susceptible genotypes. **(B)** Number of DECs for pairwise comparisons between the tolerant and susceptible genotypes for each time point (0, 6, and 12 h) for control and inoculated conditions.

For each contig, twelve pairwise comparisons were performed (Tables S3, S4). In order to identify which genes were differentially expressed in response to the *Fsp* challenge, six comparisons were performed that evaluated the expression values of genes in non-inoculated versus *Fsp*-inoculated treatments, as listed in Supplementary Table S4-comparisons 1 to 6 (Figure 1A). In order to identify which genes responded differentially to *Fsp* between the tolerant (bulked-tolerant) and susceptible (bulked-susceptible) genotypes, six additional pairwise comparisons were performed and are summarized in Supplementary Table S4-comparisons 7 to 12 (Figure 1B). The twelve pairwise comparisons resulted in the identification of 42,905 DECs out of the 185,721 contigs.

**FIGURE 2:**
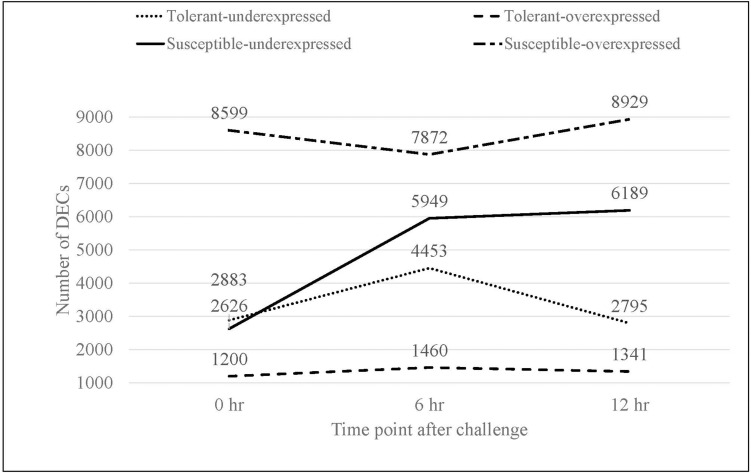
The total number of overexpressed and underexpressed DECs in the inoculated treatments for tolerant and susceptible genotypes at each time point in response to the *Fsp* challenge.

Pairwise comparisons 1 to 6 that compare non-inoculated versus *Fsp*-inoculated treatments, yielded the number of upregulated DECs obtained for each time point (Figure 2). For the *Fsp* inoculated tolerant genotypes, the number of upregulated DECs varied between 1,200 and 1,460 DECs across 0, 6-, and 12-h time points. The number of suppressed (under-expressed) DECs in the inoculated tolerant genotypes was larger (2795-4453 DECs). For the *Fsp* inoculated susceptible genotypes, the total number of upregulated DECs was 5–7 times larger than the total number for the *Fsp* inoculated tolerant genotypes (Figure 2). The number of suppressed genes in both sets of genotypes was similar at 0 h. However, for the susceptible genotypes, these numbers were 1.34 and 2.21 times larger than the tolerant genotypes at 6 and 12 h, respectively. A total of 5.7 and 5.4% of the DECs were shared across the three time points for the tolerant and susceptible genotypes, respectively (Supplementary Table S3). From the total upregulated DECs in the *Fsp* inoculated sets, 10 (0.10%), 48 (0.52%), and 12 (0.12%) DECs are shared among the tolerant and susceptible genotypes at the 0, 6, and 12-h time points, respectively (Supplementary Table S3).

### Functional Annotation of DECs and GO-Term Enrichment

From the 185,721 contigs of the master assembly, 120,132 returned positive BLAST hits when queried to the NCBI database (National Center for Biotechnology Information, 2020). A total of 4,734 contigs or 2.55% of the total contigs were annotated as proteins of unknown function or hypothetical proteins. The top BLAST hits showed similarity to *M. truncatula*, *Trifolium pratense*, *Cicer arietinum*, and *Trifolium subterraneum* with a distribution of 34.0, 16.1, 16.0, and 14.6% respectively. *P. sativum*, with a 3.6% match, was fifth in the rankings. The low percentage of hits was most likely due to the relatively small number of *P. sativum* RNAseq data represented in the NCBI database. Of the 42,905 DECs, 36,923 (86.1%) returned positive BLAST hits when aligned to the NCBI database. Interestingly, 33 contigs (0.09%) of the 36,923 hits were classified as proteins of unknown function or hypothetical proteins, which could be useful candidates for understanding the pea-*Fsp* interaction.

The GO enrichment analysis identified significant over- and under-represented GO terms for each of the three *Fsp* inoculated time points (Supplementary Table S5). GO terms related to routine DNA processes, such as DNA metabolic process, DNA biosynthetic process, and DNA integration were significantly underrepresented at different time points in the tolerant and susceptible genotypes. DNA integration and DNA metabolic process terms were underrepresented across the three times in the susceptible genotypes, but only at 0 and 6 h for the tolerant genotypes. Nucleic acid phosphodiester bond hydrolysis was also underrepresented only during certain times points in the susceptible genotypes but not in the tolerant genotypes.

**FIGURE 3:**
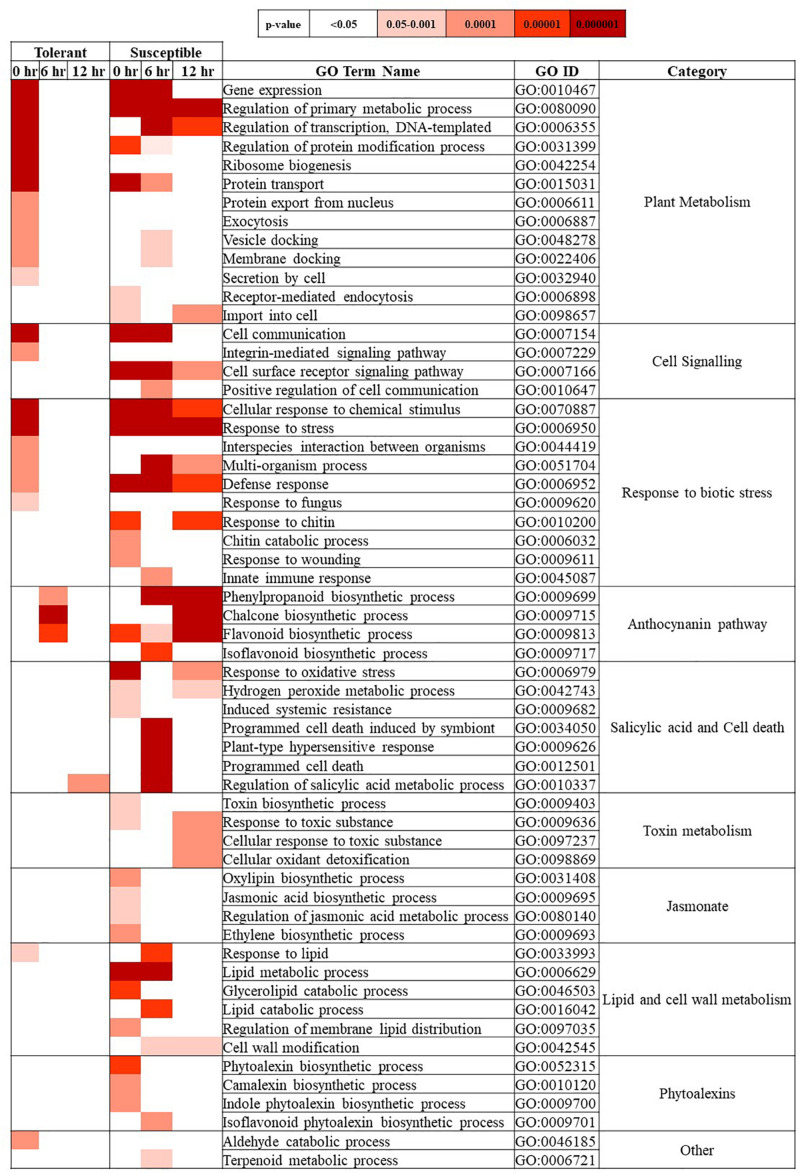
Biological process-GO terms over-represented in the *Fsp* inoculated treatment for tolerant and susceptible genotypes at 0, 6, and 12-h time points. The tone of colors in the heatmap denotes the *p*-value calculated using two-tailed Fisher’s exact test. Significantly over-represented GO terms showed a *p* > 0.05. *p*-values.

Figure 3 represents a heat map with the overrepresented biological process GO terms for each time point in the inoculated treatment for the tolerant and susceptible genotypes. These terms provide a comparative perspective of biological processes that responded to *Fsp* in the two subsets of genotypes.

### Quantitative RT-PCR Verification

**FIGURE 4:**
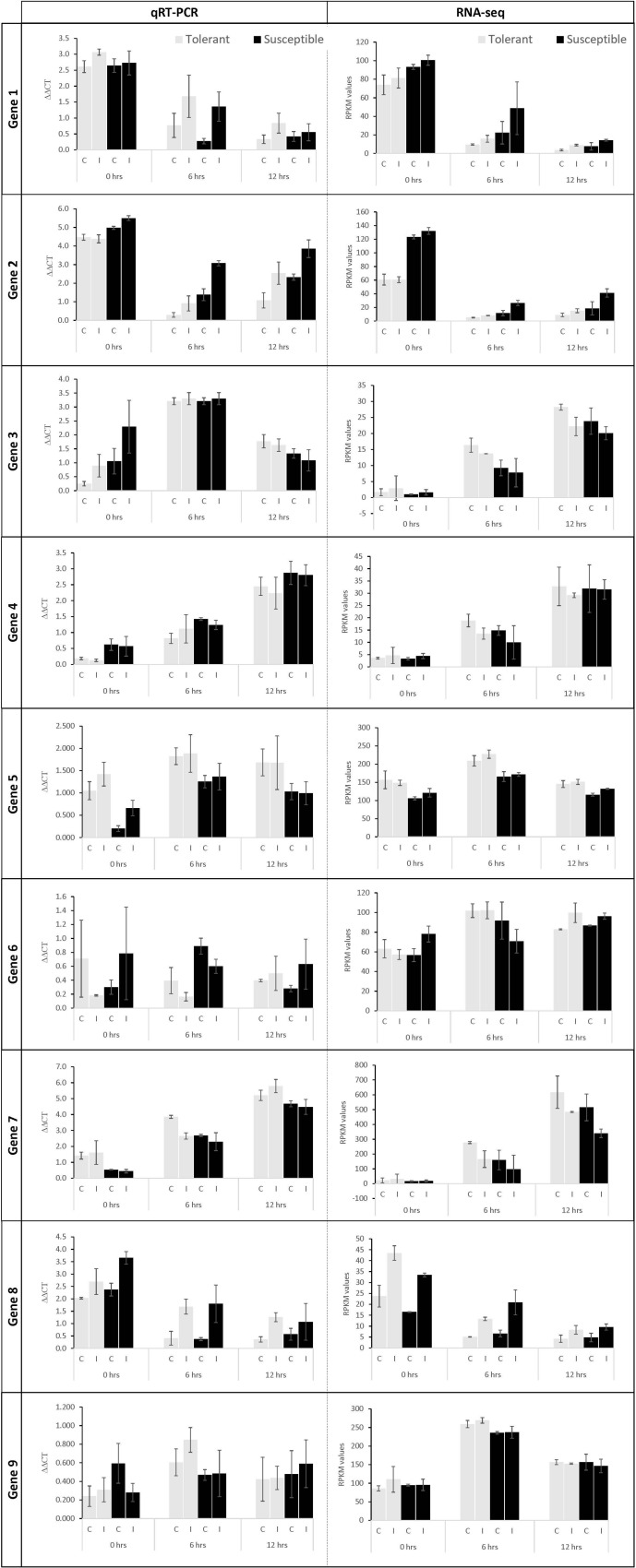
RT-qPCR validation of select genes in control (C) and Fsp inoculated (I) plants. The gray and black bars represent relative gene expression for the tolerant and susceptible genotypes, respectively. First column: RT-qPCR data show the average relative expression of three biological samples with three technical replicates each. Second column: RPKM values calculated for each gene. The error bars represent the standard error between replicates in RT-qPCR analysis. Gene 1: TRINITY_DN2419_c0_g1_i5 (BLAST accession: XM_003592027.3), Gene 2: TRINITY_DN2754_c0_g1_i11 (BLAST accession: XM_004502933.3), Gene 3: TRINITY_DN5727_c0_g1_i1 (BLAST accession: MK618561.1), Gene 4: TRINITY_DN6240_c0_g1_i9 (BLAST accession: XM_003592048.3), Gene 5: TRINITY_DN2169_c1_g1_i2 (BLAST accession: XM_004506541.3), Gene 6: TRINITY_DN1232_c0_g1_i11 (BLAST accession: XM_004504351.3), Gene 7: TRINITY_DN5529_c0_g1_i9 (BLAST accession: XM_024782286.1), Gene 8: TRINITY_DN8631_c0_g1_i1 (BLAST accession: XM_013611166.2), Gene 9: TRINITY_DN1795_c0_g1_i2 (BLAST accession: XM_004514502.3).

To verify the expression results obtained from RNAseq data, RT-qPCR analysis was performed on nine randomly selected genes at all time points for the tolerant and susceptible genotypes. The expression trends of eight out of nine genes (89%) correlated with the RPKM values indicating the robustness of RNAseq results (Figure 4). The internal reference control (GenBank accession AF1139187.1) showed invariant expression across genotypes and treatments in this RT-qPCR analysis.

### *Fsp*-Induced Differential Gene Expression

**FIGURE 5:**
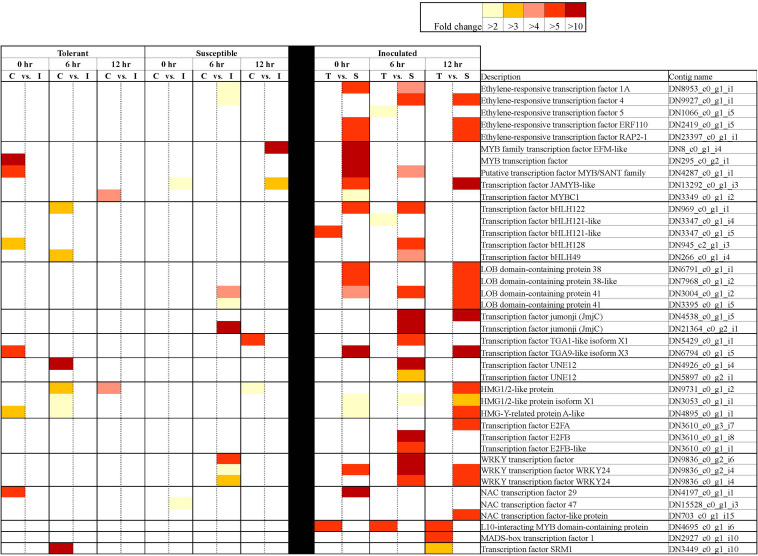
Differentially expressed transcription factors in tolerant and susceptible pea genotypes in response to *Fsp* challenge. The color key denotes fold-change. Pairwise comparisons that displayed greater than two-fold difference (*p* < 0.001) in expression were identified with a color that ranges from light yellow (fold change >2) to dark red (fold change >10).

**FIGURE 6:**
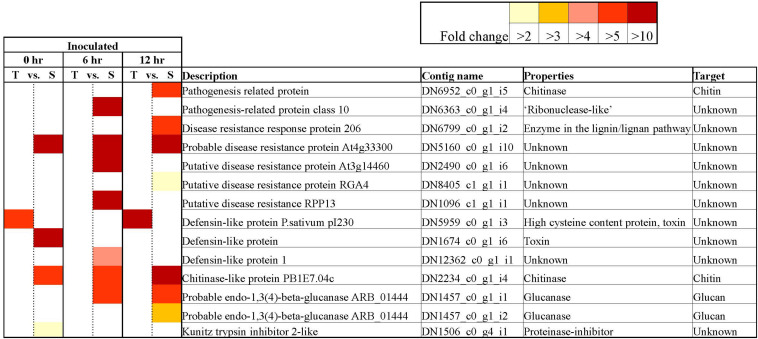
Key differentially expressed Pathogenesis-related contigs in tolerant and susceptible pea genotypes in response to *Fsp* challenge. The color key denotes fold-change. Pairwise comparisons that displayed greater than two-fold difference (*p* < 0.001) in expression were identified with a color that ranges from light yellow (fold change >2) to dark red (fold change >10).

**FIGURE 7:**
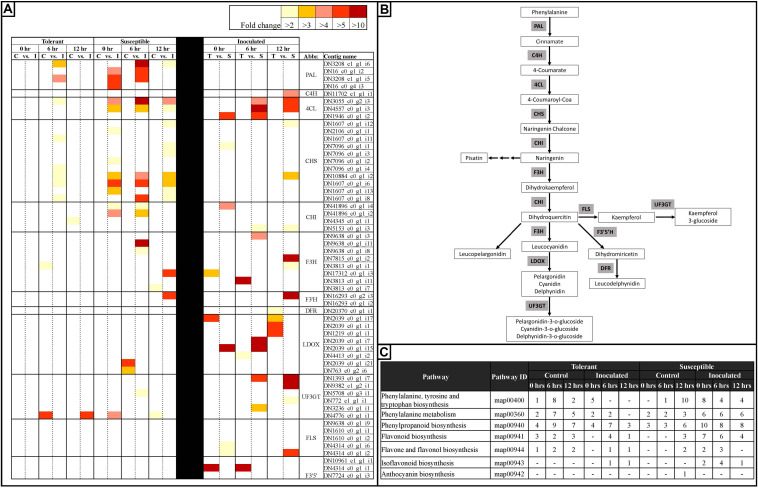
Heatmap representation of changes in the expression of genes associated with the anthocyanin biosynthetic pathway in tolerant and susceptible pea genotypes after *Fsp* challenge. **(A)** A subset of differentially expressed genes involved in the anthocyanin biosynthetic pathway in tolerant and susceptible pea genotypes after the *Fsp* challenge. The color key denotes fold-change. Pairwise comparisons that displayed greater than two-fold difference (*p* < 0.001) in expression were identified with a color that ranges from light yellow (fold change >2) to dark red (fold change >10). **(B)** Anthocyanin biosynthesis pathway (Adapted from Solfanelli et al., 2006). **(C)** KEGG pathway analysis of metabolic processes related to the anthocyanin biosynthetic pathway. Abbreviations (Abbr.) PAL, phenylalanine ammonia lyase; CHS, chalcone synthase; CHI, chalcone isomerase; F3H, flavanone-3-hydroxylase; F3′H, flavonoid 3′-hydroxylase; DFR, dihydroflavonol 4-reductase; LDOX, leucoanthocyanidin dioxygenase; UF3GT, UDP glucose-flavonoid 3-o-glucosyltransferase; FLS, Flavonol synthase; F3′5′H, Flavonoid 3′,5′-hydroxylase.

**FIGURE 8:**
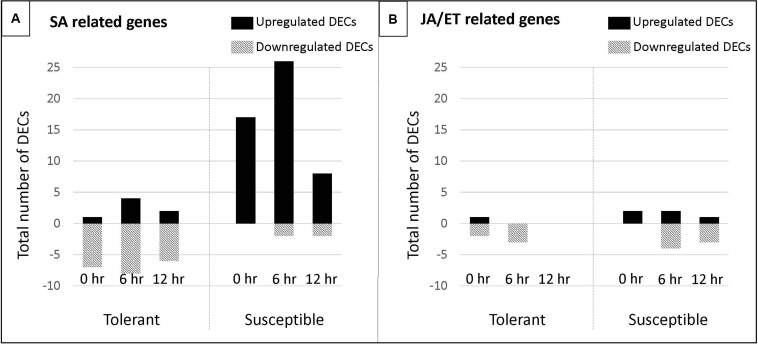
Number of DECs associated with the salicylic (SA) and jasmonate/ethylene (JA/ET) biosynthetic and signaling pathway in a pea tolerant and susceptible genotype after *Fsp* challenge. **(A)** DECs associated with the salicylic (SA) biosynthetic and signaling pathway. **(B)** DECs associated with the jasmonate/ethylene (JA/ET) biosynthetic and signaling pathway.

**FIGURE 9:**
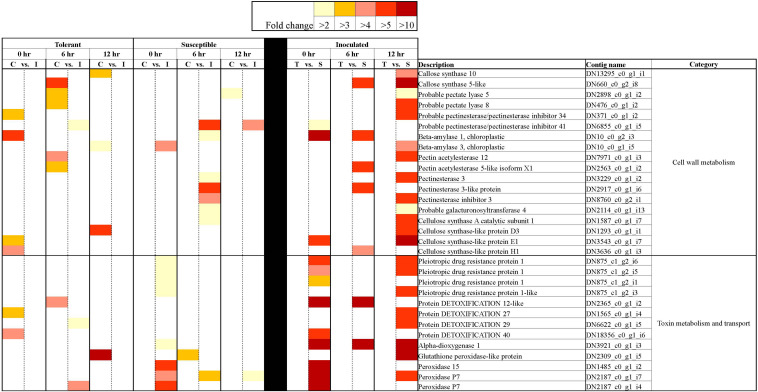
Heatmap representation of differentially expressed genes associated with cell wall metabolism, toxin metabolism and transport in tolerant and susceptible pea genotypes in response to *Fsp* challenge. The color key denotes fold-change. Pairwise comparisons that displayed greater than two-fold difference (*p* < 0.001) in expression were identified with a color that ranges from light yellow (fold change >2) to dark red (fold change >10).

A closer look at the 42,905 DECs showed that most of these genes (86.1%) returned positive BLAST hits. The genes were placed into seven broad categories for understanding their potential role in responding to *Fsp* challenge: (1) Expression of signaling-related genes (Supplementary Table S3), (2) Genes involved in transcriptional regulation (Figure 5 and Supplementary Table S3), (3) PR genes (Figure 6 and Supplementary Table S3), (4) Anthocyanin and lignin biosynthetic pathway genes (Figure 7 and Tables S3, S6), (5) Sugar metabolism (Supplementary Table S3), (6) Phytohormones (Figure 8 and Supplementary Table S3), (7) Cell wall and membrane metabolism and toxin metabolism (Figure 9 and Supplementary Table S3).

## Discussion

### Assembly of Transcriptome Data and Identification of Differentially Expressed Contigs (DECs)

The susceptible genotypes showed a larger number of upregulated DECs that represented a more diverse and higher number of transcriptionally regulated genes, in contrast to the tolerant genotypes. This higher number of upregulated DECs in the susceptible genotypes may be due to involvement of various biological processes associated with successful *Fsp* infection in pea embryonic tissue. This observation is consistent with previous studies, which showed that pathogen attack engages a broader range of pathways and a larger proportion of genes in the susceptible genotypes compared to the resistant ones (Bagnaresi et al., 2012; Zheng et al., 2013; Matic̃ et al., 2016).

Only 10 (0.10%), 48 (0.52%), and 12 (0.12%) DECs are shared among the tolerant and susceptible genotypes at the 0, 6, and 12-h time points, respectively. Therefore, the response mechanisms involved in the *Fsp* challenge were divergent between the tolerant and susceptible genotypes. The large difference in overexpressed DECs could explain the difference in tolerance between these sets of genotypes. The genes that were overexpressed in susceptible genotypes were numerically different from the ones in the tolerant genotypes.

### Functional Annotation of DECs and GO-Term Enrichment

The GO-term enrichment analysis showed that common terms related to basic plant metabolism, such as gene expression, regulation of primary metabolism, transcription, and protein synthesis were over-represented at the 0-h time point in the tolerant genotypes. Conversely, these terms were upregulated in at least two time points in the susceptible genotypes. Therefore, these results showed the tolerant genotypes may have moved toward a basal metabolic state after the 0-h time point, while the susceptible genotypes were responding to *Fsp* throughout the entire time course presented in this study.

Gene ontology terms such as ribosome biogenesis, protein export from nucleus, exocytosis, and secretion by cell were over-represented at 0 h in the tolerant genotypes. Plants are known to transport antimicrobial molecules, such as peptides and/or secondary metabolites, outside the cell to function in plant immunity (Kwon and Yun, 2014). In contrast, the susceptible genotypes seemed to be importing substances inside the cell since terms such as receptor-mediated endocytosis and import to the cell were over-represented. Endocytosis seems not only to play a role in pathogen-associated molecular patterns (PAMP)-triggered immunity and effector-triggered immunity but also in susceptibility. Vesicle endocytosis can be manipulated by pathogens and can import pathogen-derived effectors into the plant cell (Driouich et al., 1997; Kwon and Yun, 2014). In this study, while the tolerant genotypes seemed to export antimicrobial molecules potentially to counter *Fsp*, the susceptible genotypes seemed to import substances. Thus, it is hypothesized that the suppression of exocytosis mechanisms in the susceptible genotypes might block or delay the transport and release of antimicrobial substances against pathogens.

Gene ontology terms in the cell signaling, and response to biotic stress categories also showed an early and unique response at 0 h in the tolerant genotypes, while this response was present throughout the entire experiment (0, 6, and 12 h) or at later stages (6 or 12 h) in susceptible genotypes. Several studies have analyzed how pea responds to *Fsp* and the non-host pathogen *F. solani* f. sp. *phaseoli* (*Fsph*). These studies concluded that the major difference is the speed at which the pea plants react. The type of response exhibited by pea varies with the rate of induction of PR genes and other associated biochemical pathways. In the case of either *Fsph* or *Fsp* infection, the fungus releases DNAses, which localize to the host nuclei and digest the nuclear DNA (Hadwiger and Adams, 1978; Hadwiger, 2008, 2015). Fungal DNAses can also impact the nuclei in the fungal mycelia and trigger their deterioration (Hadwiger, 2008). In the case of a compatible interaction (successful infection leading to disease) between *Fsp* and pea, the slower reaction rate of the pea host allows *Fsp* to protect a small number of its nuclei from fungal DNAses. The slower reaction allows the growth of *Fsp* to resume after 12 h post-inoculation (Klosterman et al., 2001; Hadwiger, 2015). In contrast, the relatively rapid response generated in the host against *Fsph* terminates the growth of the fungi at 6 h. post-inoculation (Hadwiger, 2008, 2015). Given this information, it is hypothesized the speed of reaction to the pathogen may be one of the mechanisms of tolerance in the tolerant genotypes.

The GO terms associated with salicylic acid and cell death category were, in most cases, overrepresented only in the susceptible genotypes. Therefore, the susceptible genotypes were expected to have a more intense response to *Fsp* through induced systemic resistance, host programmed cell death, and plant-type hypersensitive response.

The GO terms associated with the production of flavanones, flavones, flavonols, proanthocyanidins, and anthocyanins showed over-representation in both genotypes. In the tolerant genotypes, these terms were only over-represented at 6 h. In the susceptible genotypes, GO terms from this category were overrepresented at the 0, 6, and 12-h time points, indicating a more intense response. GO terms associated with the jasmonate pathway, phytoalexin synthesis, toxin metabolism, and lipid metabolism were overrepresented only in the susceptible genotypes at all time points (Figure 3 and Supplementary Table S5).

### *Fsp*-Induced Differential Gene Expression

#### Expression of Signaling-Related Genes

Plants detect pathogens via host sensors known as pattern-recognition receptors (PRR), which act by detecting PAMPs and triggering PAMP-triggered immunity (PTI). Beck et al. (2012) identified three PRRs that have been proven to be specific to fungi: the Chitin Elicitor Binding Protein (CEBiP), the chitin elicitor receptor kinase I (CERK1), and the ethylene-inducing xylanase (Eix2). CEBiP and CERK1 cooperatively regulate chitin elicitor signaling to activate plant defense system (Hayafune et al., 2014). Interestingly, CEBiP was not identified in this study. Contigs corresponding to CERK1 and Eix2 genes were identified, however they were not differentially expressed in any of the genotypes.

In this study, several LRR-RLK receptors were found to be differentially expressed in response to *Fsp* challenge (Supplementary Table S3). Most of these genes were upregulated in the susceptible rather than in the tolerant genotypes. The L-type lectin-domain containing receptor kinase, proline-rich receptor-like protein kinase, cysteine-rich receptor-like protein kinase and the wall-associated receptor kinases are also upregulated in the susceptible genotypes, following the same trend as above. The activity of Mitogen-activated protein kinases (MAPK), MAPK kinases (MAPKK), and MAPKK kinases (MAPKKK) were, in most cases, upregulated in the tolerant and susceptible genotypes after the challenge with *Fsp* when compared to the control. However, the expression was, in most cases, significantly higher in the susceptible genotypes throughout the time-course when compared to the tolerant genotypes.

Two contigs identified as receptors were found to change significantly in expression after the *Fsp* challenge in the susceptible genotypes only. Contig DN1290_c0_g1_i9 and Contig DN7023_c0_g2_i5 were identified as a receptor-like cytoplasmic kinase 176 and CC-NBS-LRR resistance protein (Supplementary Table S3), respectively. The receptor-like cytoplasmic kinase 176 acts downstream of the CERK1 gene in the fungal chitin signaling pathways that mediates innate immunity responses such as reactive oxygen species generation, defense gene expression, and callose deposition (Ao et al., 2014). The CC–NBS–LRR proteins initiate a resistance response that often includes a type of cell death known as the hypersensitive response (HR) (Moffett et al., 2002). In the susceptible genotypes, contig DN1290_c0_g1_i9 was significantly overexpressed at 6 h (FC = 3.19) after the *Fsp* challenge. Expression of Contig DN7023_c0_g2_i5 was found to be lowered at 12 h (FC = −3.59) after the *Fsp* challenge. Interestingly, no change was observed in the expression of the two contigs in the tolerant genotypes. These two contigs also showed higher expression in the susceptible genotypes when compared to their expression in the tolerant genotypes. Contigs DN1290_c0_g1_i9 and DN7023_c0_g2_i5 were significantly upregulated at 0 (FC = 3.49), 6 (FC = 5.08), and 12 h (FC = 6.77), and 6 h (FC = 71.69), respectively, in the inoculated treatments of the susceptible genotypes when their expression values were compared to the tolerant genotypes. The data on the observed induction of genes coding for receptor-like cytoplasmic kinase 176 and CC-NBS-LRR resistance protein in the susceptible genotypes suggest that the pathogen likely recruits oxygen species generation, hypersensitive response, defense gene expression, and callose deposition to establish infection. It raises a question if loss of function mutation in these genes in the susceptible genotypes could confer tolerance to *Fsp*. A loss-of-function mutation in receptor-like cytoplasmic kinases and CC-NBS-LRRs genes has proven to confer tolerance to different pathogens (Lorang et al., 2007; Sweat and Wolpert, 2007; Zhang et al., 2019).

#### Genes Involved in Transcriptional Regulation

The following TFs were found to be differentially expressed between the tolerant and susceptible genotypes and/or were influenced by *Fsp* challenge: bZIP, ERF, MYB, GATA, MADS-box, NAC, PLATZ, KAN2, PosF21, WRKY, C2H2, bHLH, DIVARICATA, E2F, GLABRA, ICE, IIIB, Jumonji, PIF, RF2a, SRM1, TCP19, TGA, UNE12, and HMG. From this list of TFs, bZIP, ERF, MYB, MADS-box, NAC, WRKY, C2H2, bHLH, E2F, Jumonji, PIF, RF2a, TCP19, TGA, and HMG have been reported to be master regulators of defense responses against pathogens (Pontier et al., 2001; Vailleau et al., 2002; Dong et al., 2003; Pré et al., 2008; Isaac et al., 2009; Wang et al., 2009; Kiełbowicz-Matuk, 2012; Alves et al., 2013; Li et al., 2013; Song et al., 2013; Chandran et al., 2014; Khong et al., 2015; Li, 2015; Paik et al., 2017; Im et al., 2019). However, the involvement of following TFs in response to pathogen challenge has not been reported previously – GATA, PLATZ, KAN2, PosF21, DIVARICATA, GLABRA, ICE, IIIB, SRM1, UNE1.

In the tolerant genotypes, TFs were either not differentially expressed when the control and inoculated samples were compared, or their expression was significantly suppressed after *Fsp* challenge. In the susceptible genotypes, however, the expression of TFs remained the same or increased after *Fsp* challenge. When the inoculated treatments in the tolerant and the susceptible genotypes were compared at either time point, TFs were overexpressed in the susceptible genotypes (Figure 5). It is well documented that overexpression of certain TFs causes susceptibility to certain pathogens (Kim et al., 2006; Lai et al., 2008; Thatcher et al., 2012). Some examples include the enhanced susceptibility of the *WRKY7*-overexpressing Arabidopsis plants to *Pseudomonas syringae* infection. Overexpression of the AtWRKY4 gene in Arabidopsis enhances susceptibility toward the biotrophic bacterium *P. syringae* (Lai et al., 2008). In a disease screen with *Fusarium oxysporum*, it was found that disruption of the *LATERAL ORGAN BOUNDARIES* (*LOB*) *DOMAIN* (*LBD*) TF led to increased resistance to *F. oxysporum* root-rot disease in *Arabidopsis thaliana*.

From the extensive list of differentially expressed TFs identified in this study, only the HMG A has been previously shown to participate during pea-*Fsp* interaction (Hadwiger, 2008). HMG A has been shown to complex with promoter areas of Disease-resistance response (DRR) genes in pea after *Fsp* infection (Klosterman et al., 2003). Transient expression experiments implicate the HMG-I/Y abundance in the down-regulation of DRR206 gene expression in pea (Klosterman et al., 2003). In this study, contigs DN4895 and DN12030 were identified as HMG-I/Y genes. Interestingly, the expression of the DN4895 contig decreases significantly at 0 h (FC = −3.59) and 6 h (FC = −2.65) after *Fsp* challenge in the tolerant genotypes. On the other hand, the expression of this contig increased significantly at 6 h (FC = 3.51) after *Fsp* challenge in the susceptible genotypes. Furthermore, the expression of contig DN4895 was significantly higher under inoculated conditions at 0 h (FC = 2.56) and 12 h (FC = 7.91) for the susceptible genotypes compared to these expression values in the tolerant genotypes. Contig DN12030 is also significantly overexpressed under inoculated conditions in the susceptible genotypes at 0 h (FC = 3.23) and 12 h (FC = 5.70). It is plausible that HMG-I/Y mediates the reduction of expression of defense-related genes in the susceptible genotypes; however, this assumption will need to be further evaluated.

#### Pathogenesis-Related (PR) Genes

The inoculation of the tolerant and susceptible genotypes with *Fsp* generated changes in the expression of PR genes. All the PR protein encoding genes that were identified in this experiment were overexpressed in the susceptible genotypes over the tolerant genotypes except one (Figure 6). The contig DN5959_c0_g1_i3 was identified as a defensin named *P. sativum* pI230 mRNA (*e*-value: 0.0, percentage identity: 97.23%). The expression of the DN5959_c0_g1_i3 contig was significantly higher in the tolerant genotypes over the susceptible genotypes under control conditions at 0 (FC = −69.21) and 6 h (FC = −151.78), and under inoculated conditions at 0 (FC = −7.93) and 12 h (FC = −43.5). The pI230 mRNA is the precursor for the DRR230 protein, which is a disease resistance response protein identified previously in *P. sativum*. DRR230 defensin was first identified in pea pods in response to infection by *Fsp* (Chiang and Hadwiger, 1991). This defensin was also found to co-localize with a major QTL (*mpIII-4*) involved in resistance to *Mycosphaerella pinodes* in pea (Prioul-Gervais et al., 2007). DRR230 gene does not co-localize with any of the major QTLs identified by Coyne et al. (2019).

Disease-resistance response 230 was isolated by Almeida et al. (2000) and characterized as a small cysteine-rich polypeptide. Almeida et al. (2000) also determined that DRR230 is very effective as a fungal growth inhibitor against *Aspergillus niger*, *Aspergillus versicolor*, *Fsph*, and *Neurospora crassa*. The specific function of DRR230 is not yet known, however plant defensins form a characteristic structure known as the cysteine-stabilized α/β motif, a feature that is also shared by several toxins from insects, scorpions, honeybees, and spider venoms (Hadwiger, 2008). The pea DRR230 was overexpressed in canola, and these plants inhibited were significantly more resistant to *Leptosphaeria maculans*, a hemibiotrophic fungus (Wang et al., 1999). The transcriptome analysis presented in this study reinforces recent and preceding studies that suggest that DRR230 may play a key role in resistance or tolerance to *Fsp* induced root rot.

#### Anthocyanin and Lignin Biosynthetic Pathway Genes

The germplasm utilized in this study consisted of white-flowered lines that present partial tolerance to *Fsp*. RNAseq analysis showed that the white-flowered lines contain a large set of DECs that participate in the anthocyanin biosynthetic pathway (Figure 7). Genes coding for enzymes in the phenylalanine ammonia lyase (PAL) to chalcone isomerase (CHI) biochemical pathway were upregulated in the inoculated treatments in both the tolerant and susceptible genotypes. Furthermore, some of these enzymes were either overexpressed in the susceptible genotypes or were expressed at a similar level between the tolerant and susceptible genotypes (Figure 7).

The expression patterns were more variable for genes coding for enzymes from the flavanone-3-hydroxylase (F3H) to leucoanthocyanidin dioxygenase (LDOX), as well as for the flavonoid 3′,5′-hydroxylase (F3′5′H), UDP glucose-flavonoid 3-o-glucosyltransferase (UF3GT), and flavonol synthase (FLS) enzymes (Figures 7A,B). Some isoforms of F3H were overexpressed in the susceptible genotypes, but also some other isoforms were overexpressed in the tolerant genotypes. F3H, UF3GT, and FLS were upregulated in the susceptible genotypes, but most or all isoforms of LDOX, and F3′5′ were upregulated in the tolerant genotypes. In the susceptible genotypes, expression of some isoforms of LDOX was suppressed after challenge with *Fsp* (Figures 7A,B).

Figure 7C also shows the differential expression of genes at each time point, treatment, and genotype in this study. Early steps in the phenylpropanoid pathway such as phenylalanine biosynthesis, phenylalanine metabolism, phenylpropanoid biosynthesis, flavonoid biosynthesis, flavone and flavonol biosynthesis are active in the control replicates of the tolerant genotypes at the three times points. In the susceptible controls, fewer, or none of the enzymes were overexpressed. This observation suggests that the tolerant genotypes had a higher level of expression of genes in the phenylpropanoid pathway under the basal conditions and, therefore, it was potentially better prepared to defend against *Fsp*.

Coyne et al. (2019) reported a significant QTL (*Fsp-Ps2.1*) that accounts for 44.4 to 53.4% of the phenotypic variance for resistance to *Fsp* and this QTL shows a confidence interval of 1.2 cM. *Fsp-Ps2.1* was mapped within the interval of the pigmented flower/anthocyanin pigmentation gene called as gene A in that study. However, the gene was mapped in a white flower cross. One hypothesis is that the resistance gene(s) may have been linked in the genome with gene A in the pigmented lines. The white-flowered, resistant parent may have been obtained through a linkage break between *Fsp-Ps2.1* and *A*. Alternatively, a metabolite, possibly a colorless one, in the anthocyanin pathway might be the one that provides this resistance. Fine mapping or gene knockouts are necessary to test this hypothesis.

The transcriptome data, generated in this study, was aligned via BLAST against the *Fsp-Ps2.1* ± 1.2 cM sequence (Supplementary Table S6). BLAST analysis returned 500 positive blast hits when queried to the transcriptome data. A total of 156 contigs showed differential expression after *Fsp* challenge or when the tolerant and susceptible genotypes expression values are compared (Supplementary Table S6). A total of 22 of the 156 contigs were annotated as proteins of unknown function or hypothetical proteins. Only the contig TRINITY_DN4823 was identified as a disease-related gene, soyasaponin III rhamnosyltransferase. However, this contig was overexpressed in the susceptible genotypes when compared to the tolerant genotypes. No genes associated with pigmentation were identified in the *Fsp-Ps2.1* region during this analysis.

Legumes contain the isoflavone synthase enzyme, which redirects phenylpropanoid pathway intermediates, such as naringenin, to the synthesis of isoflavonoid phytoalexins (Sreevidya et al., 2006). The isoflavonoid phytoalexins are low molecular weight antimicrobial compounds (Smith and Banks, 1986; Jeandet et al., 2014). Pisatin is an extensively studied phytoalexin from pea. In pea, the presence of *Fsp* increases the production of pisatin (Hadwiger and Beckman, 1980). The 6a-hydroxymaackiain-3-O-methyltransferase, enzyme directly upstream from the synthesis of pisatin, was expressed in both the tolerant and susceptible genotypes but overexpressed only in the susceptible genotypes at 0 h. Degradation of pisatin is an important mechanism by which *Fsp* resists pisatin and a crucial factor in the pathogenicity of *Fsp* in pea (Mackintosh et al., 1989). However, based on the results in this and previous studies, pisatin does not seem to play a role in the tolerance to *Fsp* (Mackintosh et al., 1989; Hadwiger, 2008).

The lignin biosynthetic pathway involves the central phenylpropanoid biosynthetic pathway. Genes involved in the lignin biosynthetic pathway, such as PAL, 4CL, trans-cinnamate 4-monooxygenase (C4M), and caffeoyl-o-methyltransferase (COMT), were overexpressed upon *Fsp* inoculation in both genotypes but at a significantly higher level and more consistently in the susceptible genotypes. It is well documented that the lignin biosynthetic pathway produces lignin rapidly in response to cell wall structure perturbations (Caño-Delgado et al., 2003; Tronchet et al., 2010; Sattler and Funnell-Harris, 2013; Miedes et al., 2014). Therefore, it seems the susceptible genotypes are responding to the aggressive *Fsp* invasion with a late and futile effort that involves a higher level of lignin synthesis and deposition.

#### Sugar Metabolism

This study identified DECs involved in sugar transport such as sugar transporter ERD6-like 6, sugar carrier protein C-like, sucrose transport protein SUC3, sugar transport protein 13, probable alkaline/neutral invertase D, bidirectional sugar transporter SWEET2-like, and invertase inhibitor-like protein. These sugars transporters are upregulated in the susceptible genotypes (Supplementary Table S3).

Certain pathogens are known to modulate the expression and activity of sugar transporters during their interaction with the plant host. Bacterial and fungal pathogens induce the overexpression of different sugar efflux transporters, such as the SWEET genes; this overexpression results in sucrose accumulating in the apoplast for use in pathogen nutritional gain and growth (Chen et al., 2010; Lanubile et al., 2015). The results of this study are in concordance with the literature, suggesting that the active mobilization of sucrose in the *Fsp*-inoculated susceptible genotypes supported successful infection by *Fsp*. Of all the DECs identified in the susceptible genotypes, 78% (25 genes) were overexpressed, and only 22% (7 genes) were suppressed after the *Fsp* challenge. In the tolerant genotypes, 15% (3 genes) were overexpressed, and 85% (17 genes) were suppressed after the *Fsp* challenge. These data would support the scenario explained previously; *Fsp* is either manipulating sugar metabolism or taking advantage of the active mobilization of sucrose in the susceptible genotypes.

#### Phytohormones

A large group of DECs were identified that were involved in the synthesis and signaling of salicylic acid (SA), jasmonic acid (JA), and ethylene (ET). The GO enrichment analysis also showed overrepresented GO terms related to the synthesis and signaling of these three hormones. From this set of DECs, a few were overexpressed in the tolerant genotypes, but the vast majority were overexpressed in the susceptible genotypes after the *Fsp* challenge.

It is generally assumed that the trophic nature of the pathogen determines which signal transduction pathway (SA or JA/Et) becomes activated in the plant host (Halim et al., 2006). Biotrophic pathogens generally elicit the defense responses via the salicylic acid signaling pathway, while necrotrophs activate a JA-dependent defense response (McDowell and Dangl, 2000; Dangl and Jones, 2001; Thomma et al., 2001; Van Wees et al., 2003; Grant and Lamb, 2006; Halim et al., 2006; Trusov et al., 2009; El Rahman et al., 2012). SA production promotes cell death, and that in turn promotes additional SA production. SA-signaling travels through the plant activating systemic acquired resistance (SAR) (Glazebrook, 2005). Necrotrophic pathogens have been shown to hijack plant defense responses to produce SA to further promote disease development. The necrotrophic fungi, *Botrytis cinerea*, and *Alternaria solani* use the SA-signaling pathway to exacerbate the disease in tomato (El Rahman et al., 2012). Both pathogens use the SA-signaling pathway through NPR1, a master regulator of SA signaling, and TGA1a TF to promote disease development in tomato. NPR1 and TGA1a suppress the expression of proteinase inhibitors, which in turn suppress the expression of two JA-dependent defense genes (El Rahman et al., 2012).

In this study, differential expression of genes associated with SA synthesis and signaling, cell death and HR in both the tolerant and susceptible genotypes was observed. However, when comparisons were made between the tolerant and susceptible genotypes, these DECs were observed to be overexpressed in the susceptible genotypes. The contig DN5429 in this study was identified as TGA1.a transcription factor (involved in SA signaling) and its isoform DN5429_c0_g1_i1 was overexpressed 6.82-fold in the susceptible genotypes over the tolerant genotypes. These are important observations, at least at the gene expression level, since the current understanding portends that overexpression of SA-related genes should not be observed in interactions between *Fsp* and pea, as cell death in plant hosts does not limit pathogen growth (Glazebrook, 2005). SA-dependent responses and SAR are not predicted to play a role in tolerance against *Fsp*, whereas responses mediated by JA and ET are expected to do so.

The overexpression of genes associated with JA/ET synthesis and signaling also happens in both the tolerant and susceptible genotypes. When comparisons were made between the tolerant and susceptible genotypes, 22 DECs (80% of JA/ET-associated genes) were observed to be overexpressed in the susceptible genotypes. Based on these data, it is difficult to draw conclusions on the effect of SA and/or JA/ET in the response of the tolerant and susceptible genotypes to *Fsp*. However, a comparison was made using the number of DECs associated with the SA and JA/ET biosynthetic and signaling pathway in the tolerant and susceptible genotypes after the *Fsp* challenge (Figure 8). The susceptible genotypes showed an upsurge in the overexpression of genes related to SA biosynthesis and signaling (Figure 8). Alternatively, in the tolerant genotypes, the majority of genes related to SA biosynthesis and signaling were suppressed after the *Fsp* challenge. These changes related to the SA-pathway genes in the susceptible genotypes might have (1) deteriorated the action of the JA-signaling pathway, (2) increased the cell death, and therefore, (3) facilitated successful infection by *Fsp*. Genetic mapping or gene knockouts are needed to evaluate the involvement of the SA-signaling pathway in the development of root rot disease caused by *Fsp* in the susceptible genotypes. Some potential targets for gene knockout experiments would be NPR1 and TGA1.a.

#### Others: Cell Wall and Membrane Metabolism and Toxin Metabolism

Cell wall and membranes play important roles in plant defense as they act as a barrier that prevents pathogen invasion (Vorwerk et al., 2004). In this study, genes related to the cell wall and membrane modification, and callose deposition, were mostly upregulated in the susceptible genotypes (Figure 9). The genes for cell membrane transporters, proteins that work on the detoxification (antiporter activity) of substances, and proteins that break down toxins accumulated in the plant host were also overexpressed in the susceptible genotypes (Figure 9). Interestingly, after the *Fsp* challenge, the majority of genes associated with cell wall metabolism, toxin metabolism and transport were suppressed in the tolerant genotypes, while they were overexpressed in the susceptible genotypes (Figure 9). These responses, at least at the gene expression level, indicate that the response of the susceptible genotypes was delayed as the pathogen had already infested the tissues, and therefore, the host made a futile effort in response to the pathogen attack. Most likely, the tolerant genotypes already possessed physical and biochemical barriers, and the expression of the genes related to these pathways were actually being suppressed.

## Conclusion

The time course RNAseq results presented in this study provided a comprehensive insight into the transcriptomic changes that accompany *Fsp* infection in tolerant and susceptible *P. sativum* genotypes. Eight different pea genotypes used in this study represent four most tolerant and four most susceptible genotypes identified in a preceding study based on root disease severity (RDS) index (Bodah et al., 2016). While phenotypically they were either tolerant or susceptible, four genotypes in each category represent different parentage. It is hypothesized that each of the genotypes possesses different tolerance or susceptible alleles. The identification of several key disease-related genes including, DRR230 (Chiang and Hadwiger, 1991), a known *Fsp*-tolerance gene, is indicative that a complementary approach used in this study could be utilized for other self-pollinating species for the identification of stimulus-responsive genes.

The observed changes in the expression of genes are associated with various physiological and biochemical processes that are known to be involved in plant disease response against pathogens. *Fsp* challenge produced a more intense and diverse overexpression of genes, across the entire time-course, in the susceptible genotypes compared to the tolerant genotypes. This type of response is hypothesized to be related to the speed at which the pathogen infestations advances in the susceptible genotypes and the preexisting level of disease-preparedness in the tolerant genotypes. The transcriptomic effort demonstrated by the susceptible genotypes seems futile and lacked key specific responses that were present in the tolerant genotypes. In contrast, the tolerant genotypes showed a fine-tuned response: fewer changes in the expression of defense-related genes that helps preserve energy and a faster reset to a basal metabolic state.

This RNAseq analysis helped identify alternate strategies and potential genes that could be evaluated to confer improved tolerance against root rot in *P. sativum*. Specific genes or pathways that might have a key role in tolerance or susceptibility to *Fsp* are as follows: receptor-like cytoplasmic kinase 176, CC-NBS-LRR resistance protein, *WRKY7* TF, WRKY4 TF, *LBD* TF, HMG A TF, anthocyanin biosynthetic pathway, SWEET genes, JA/ET-signaling pathway, cell death, NPR1, and TGA1.a. SA-signaling genes, and most importantly, the DRR230 protein. Functional characterization of these genes is expected to provide mechanistic information regarding pea-*Fsp* interaction, as well as gene targets for breeding. Since *Fsp* tolerance is a polygenic trait, pyramiding *Fsp* tolerance into pea cultivars is laborious but remains the most sustainable option, and it can be pursued rapidly via molecular breeding (Bodah et al., 2016) or gene editing (Ghogare et al., 2019). The rapid development of *Fsp* tolerant pea cultivars is critical for reducing yield losses and address the increasing demand for pea-derived protein for human nutrition.

## Data Availability Statement

The raw sequencing data used for RNAseq analysis is publicly available from the NCBI Sequence Read Archive (SRA, https://www.ncbi.nlm.nih.gov/sra) under the accession number PRJNA630497.

## Author Contributions

AD, RS, and BW-B designed the study. AD supervised the study. BW-B and EB performed the experiments and generated the data. BW-B and GN analyzed the data. LP provided the tolerant pea genotypes and *Fsp* isolates. All authors read and approved the final manuscript.

## Conflict of Interest

The authors declare that the research was conducted in the absence of any commercial or financial relationships that could be construed as a potential conflict of interest.
