# Supplementary material for: Identification of Fusarium solani f. sp. pisi (Fsp) Responsive Genes in Pisum sativum
Source: Front Genet. 2020 Aug 18;11:950. doi: 10.3389/fgene.2020.00950 (PMC7461991; doi:10.3389/fgene.2020.00950)
Supplement: Supplementary file 1 [file Table_1.PDF]

Table S1. Selected genes, list of primers, annealing temperatures and amplicon sizes for RT-qPCR validation.

Contig name	Blast Analysis			Primer name	Sequence 5'-->3'	Annealing Temperature (º C)	Size (bp)
	Accession	Gene name	E-value				
DN2419	XM_003592027.3	PREDICTED: Medicago truncatula ethylene-responsive transcription factor ERF110 (LOC11405418), mRNA	0.0	BWqPCR_1_fwd	GGAGGAGGATAGCCACCAGT	64.3	122
				BWqPCR_1_rev	CCTGGTCAGCAAATGGAGTT	64	
DN2754	XM_004502933.3	PREDICTED: Cicer arietinum aspartic proteinase Asp1-like (LOC101502257), mRNA	0.0	BWqPCR_2_fwd	TCTCGGGTGCTACTGAGAAA	63.5	114
				BWqPCR_2_rev	CCAACCGATGAAGAGTTGGT	63.9	
DN5727	MK618561.1	Lathyrus sativus C2H2-like zinc finger protein mRNA, partial cds	0.0	BWqPCR_3_fwd	TGTGCTGTGGAGATGGAAGA	64.3	117
				BWqPCR_3_rev	GGAAAGAGAGGGAAAATCATTG	62.6	
DN6240	XM_003592048.3	PREDICTED: Medicago truncatula uncharacterized LOC11405420 (LOC11405420), mRNA	0.0	BWqPCR_4_fwd	GATGGTGAGTGCAAAGCAAA	63.9	143
				BWqPCR_4_rev	AAGCATCAAAAGACTCGGACA	63.7	
DN2169	XM_004506541.3	PREDICTED: Cicer arietinum putative E3 ubiquitin-protein ligase XBAT31 (LOC101496789), mRNAE3 ubiquitin-protein ligase xbat31-like	6.00E-148	BWqPCR_5_fwd	TGGAAGGATCACTGGTCACA	64.4	126
				BWqPCR_5_rev	GCAAGTGTAAACGCCATTCA	63.6	
DN1232	XM_004504351.3	PREDICTED: Cicer arietinum stress-associated endoplasmic reticulum protein 2-like (LOC101506542), mRNA	1.00E-118	BWqPCR_6_fwd	AAAGGAAAAGACTACCCTGTTGG	63.2	101
				BWqPCR_6_rev	CCACTGGTTGCTGTCCTGAT	65.2	
DN5529	XM_024782286.1	PREDICTED: Medicago truncatula cysteine synthase, chloroplastic/chromoplastic (LOC11443458), mRNA	2.00E-114	BWqPCR_7_fwd	TCTCTGGCCCAGTATTCTCA	62.4	111
				BWqPCR_7_rev	CAGTTCGAAAAGCCGAAGAA	64.2	
DN8631	XM_013611166.2	GDP-l-galactose phosphorylase PREDICTED: Medicago truncatula G-type lectin S-receptor-like serine/threonine-protein kinase At1g34300 (LOC25482580), mRNA	0.0	BWqPCR_8_fwd	ATTTTGGATTGGCGAAACTG	63.6	120
				BWqPCR_8_rev	CGGCCTTTGAAGTTATTGGA	63.7	
DN1795	XM_004514502.3	PREDICTED: Cicer arietinum GDP-L-galactose phosphorylase 2-like (LOC101497835), mRNA	0.0	BWqPCR_9_fwd	TTGCAGTCTTATATTGGGGTTACA	63.1	100
				BWqPCR_9_rev	GATCTACACGGCCTCATTGC	64.5	
DN8314	AF139187.1	Pisum sativum root border cell-specific protein (BRD13) mRNA, complete cds	0.0	BWqPCR_Ctrl_fwd	TGCAGTTGCTGAAGTGTTCC	64.1	128
				BWqPCR_Ctrl_rev	GTAACGAAGGGTTGCGTGAT	63.8	
