# Supplementary material for: Identification of Fusarium solani f. sp. pisi (Fsp) Responsive Genes in Pisum sativum
Source: Front Genet. 2020 Aug 18;11:950. doi: 10.3389/fgene.2020.00950 (PMC7461991; doi:10.3389/fgene.2020.00950)
Supplement: Supplementary file 2 [file Table_2.PDF]

Table S2. Summary of Illumina reads and mapping results from RNA-seq analysis for each library generated in this study.

Library ID	Genotype (bulk)	Condition	Biological replicate	Number of raw reads	mean _Q	Number of reads after trimmed	Percentage trimmed (%)	Contig genes mapped	Contig genes mapped percentage (%)	Average length of contigs
P.sativum_S1	Tolerant	0hr Control	1	21,087,280	34.69	14,668,172	30.44	125,240	67.44	1,526.30
P.sativum_S2			2	29,751,755	34.49	20,500,575	31.09			
P.sativum_S5		0hr Inoculated	1	17,591,660	34.22	12,025,245	31.64	103,377	55.66	1,651.56
P.sativum_S6			2	38,325,318	34.31	26,156,099	31.75			
P.sativum_S9		6hr Control	1	24,060,774	34.23	16,316,996	32.18	127,056	68.41	1,561.63
P.sativum_S10			2	36,645,962	34.68	25,483,911	30.46			
P.sativum_S13		6hr Inoculated	1	33,314,160	34.35	22,806,162	31.54	110,964	59.74	1,665.00
P.sativum_S14			2	45,975,152	34.50	31,791,950	30.85			
P.sativum_S17		12hr Control	1	30,568,144	34.57	21,079,674	31.04	126,569	68.15	1,564.87
P.sativum_S18			2	37,766,216	34.54	26,055,362	31.01			
P.sativum_S21		12hr Inoculated	1	38,657,700	34.50	26,658,083	31.04	102,382	55.13	1,738.31
P.sativum_S22			2	21,378,038	34.26	18,145,808	15.12			
P.sativum_S3	Susceptible	0hr Control	1	22,860,375	34.12	16,910,696	26.03	123,269	66.37	1,532.24
P.sativum_S4			2	21,695,910	34.48	14,958,281	31.05			
P.sativum_S7		0hr Inoculated	1	35,432,148	34.45	24,358,895	31.25	137,378	73.97	1,449.52
P.sativum_S8			2	40,203,272	34.52	27,819,393	30.80			
P.sativum_S11		6hr Control	1	35,785,070	34.88	25,115,168	29.82	134,800	72.58	1,506.69
P.sativum_S12			2	27,813,826	34.43	19,097,253	31.34			
P.sativum_S15		6hr Inoculated	1	42,437,824	34.28	28,918,745	31.86	140,566	75.69	1,446.26
P.sativum_S16			2	39,370,422	34.00	26,476,697	32.75			
P.sativum_S19		12hr Control	1	63,685,406	34.62	44,134,320	30.70	141,530	76.21	1,467.68
P.sativum_S20			2	56,070,996	34.71	38,889,470	30.64			
P.sativum_S23		12hr Inoculated	1	34,620,388	34.86	24,188,558	30.13	132,009	71.08	1,531.62
P.sativum_S24			2	50,891,022	34.75	35,387,679	30.46			
