# Supplementary material for: Identification of Fusarium solani f. sp. pisi (Fsp) Responsive Genes in Pisum sativum
Source: Front Genet. 2020 Aug 18;11:950. doi: 10.3389/fgene.2020.00950 (PMC7461991; doi:10.3389/fgene.2020.00950)
Supplement: Supplementary file 3 [file Table_3.DOCX]

Table S3: Master Transcriptome Assembly

File type: xslx

Size 267 Mb

Please access here: <https://www.biorxiv.org/content/10.1101/2020.05.12.091892v1.supplementary-material>

Frontiers does not allow transfer of files larger than 30 Mb.
